# Aqueous Electrodeposition of SmCo Alloys: II. Direct Current Studies

**DOI:** 10.3389/fchem.2021.694726

**Published:** 2021-09-01

**Authors:** Jei C. Wei, Morton Schwartz, Ken Nobe, Nosang V. Myung

**Affiliations:** ^1^ University of California at Los Angeles, Los Angeles, CA, United States; ^2^ University of Notre Dame, Notre Dame, IN, United States

**Keywords:** electrodeposition, samarium cobalt, magnetic thin films, glycine, aqueous

## Abstract

Previously, we reported the aqueous electrodeposition of rare earth - iron group alloys. A key factor was the complexation of the metal ions with various coordination compounds (*e.g.,* aminoacetic acids), without which only the ferrous metal and rare earth hydroxides/oxides are deposited. In this work, samarium cobalt (SmCo) alloys were synthesized using direct current (DC) aqueous electrodeposition. The basic electrolyte solution consisted of 1 M samarium sulfamate, 0.05 M cobalt sulfate, and 0.15 M glycine, resulting in deposits containing >30 at% Sm at 60°C with current density of 500 mA/cm^2^. Supporting electrolytes (*i.e.,* ammonium salts) decreased the Sm content in the deposit. Crystallinity of deposited films altered from nanocrystalline to amorphous as the Sm content increased. Deposits with high Sm content (32 at%) became isotropic with reduction in magnetic saturation (M_s_) and coercivity (H_c_). A deposition mechanism involving stepwise reduction of the complexed Sm-Co ions by depositing hydrogen atoms was proposed.

## Introduction

High-performance permanent magnets such as samarium-cobalt (SmCo) and neodymium-iron-boron (NdFeB) alloys are playing an increasingly prominent role in miniaturizing electrical and electronic machines and devices. Although the rare earth-transition metals (RE-TM) alloys are substantially more expensive than the hard, magnetic ferrites, their superior magnetic properties drive the RE-TM permanent magnets’ growing usage (Strnat and Strnat, 1991). A sharp decline in their manufacturing costs would lead to an increasingly dominant position in worldwide applications of nano- and micro-scale systems.

Compared to SmCo, NdFeB permanent magnets (PM) have a higher energy product ((BH)_max_) and coercivity (H_c_), but a lower Curie temperature (T_C_) and chemical stability in aggressive environments. As a result, SmCo PMs have application in high temperature and aggressive environments such as those encountered by military and aeronautical / aerospace systems (du Trémolet Lacheisserie et al., 2002). So far, fabrication of nanostructured SmCo alloys have been restricted to physico-chemical deposition methods. Therefore, development of an aqueous electrodeposition process would dramatically reduce manufacturing costs (Dini, 1993).

In a series of preliminary studies, we reported on the aqueous electrodeposition of alloys of RE mischmetals, La, Ce, Nd, Gd and Sm with the iron group metals (e.g., Ni, Co, and Fe). The key factor is the complexation of the metal ions with aminocarboxylates (Chen et al., 1996; Myung et al., 1999; Schwartz et al., 1999; Schwartz et al., 2004; Wei et al., 2006; Wei et al., 2008; Wei et al., 2009). The present work reports on the aqueous DC electrodeposition of SmCo alloys using parallel electrodes. The solution constituents and compositions as well as the deposition variables were selected as a result of preliminary parametric studies using Hull Cells (HC) (Wei et al., 2008).

## Experimental

The electrodeposition cell consisted of two parallel electrodes (brass cathode, 2 × 2 cm, and a platinum anode, 3 × 6 cm), which were 4 cm apart. A shielding panel with a 2 cm by 2 cm window was inserted equidistant between the electrodes to provide a more uniform current distribution. A saturated calomel electrode (SCE) measured the cathode potential. A potentio/gavalno-stat (EG & G 273) served as the power source with a coulometer measuring charge (50 C). Solution volume was kept at 240 ml. The basic solution consisted of 1 M Sm sulfamate, 0.05 M Co sulfate, and 0.15 M glycine, unless otherwise noted. The plating conditions were varied within the following ranges: current density from 2 to 500 mA/cm^2^, temperature from 25 to 60°C, pH range from 2 to 6. The solutions were not agitated during electrodeposition.

Prior to plating, the brass cathode was mechanically cleaned by immersing in 0.1 M NaOH, rinsing with deionized (DI) water, dipping in 10 vol. % HCl (30 s) and then rinsed with DI water. The plated cathodes were rinsed and dried with nitrogen. Disk specimens (0.64 cm diameter) were fabricated for analysis and characterization.

Sm and Co contents in the deposits were determined by energy dispersive X-ray spectroscopy (EDS). Co content was measured separately by atomic absorption spectrophotometry (AAS, Perkin Elmer). Deposit structure, crystal orientation, phase identification and grain size were determined by powder X-ray diffraction (XRD). Deposit surface morphology and microstructure were observed with scanning electron microscopy (SEM). Magnetic properties were determined by a vibrating sample magnetometer (VSM, Digital Measurement Systems Model 1660) with an applied magnetic field scanning between −10 and +10 KOe. In-phase (//) and perpendicular (⊥) measurements represent the field applied to the specimen’s plane, respectively. The deposit magnetic properties were obtained from BH loops. All measurements and data reported were on deposits with metallic appearance, unless otherwise noted. CD_max_ is the maximum current density, beyond which deposits appeared non-metallic. Minimum duplicate runs were performed.

## Results and Discussion

**FIGURE 1:**
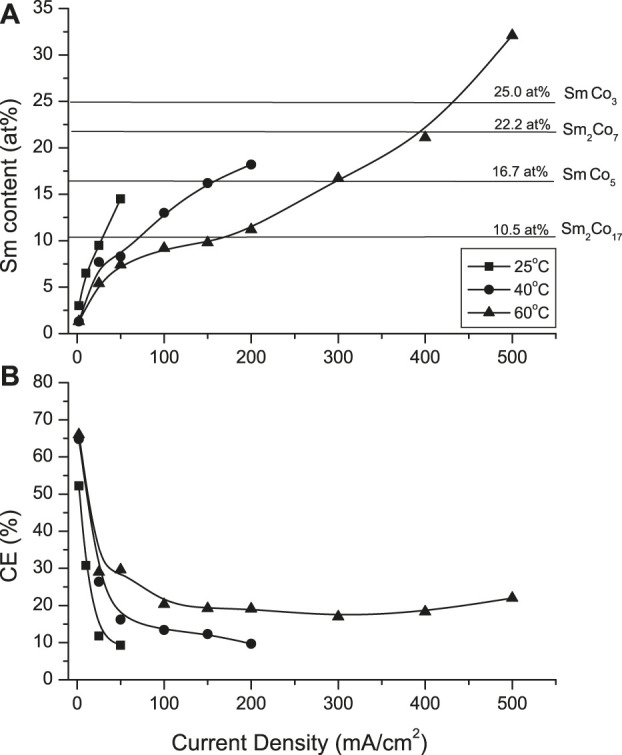
Effect of current density and solution temperature on **(A)** deposit samarium content and **(B)** current efficiency (CE).

Confirming trends of the Hull Cell (HC) studies (Wei et al., 2008), the deposit Sm content increased with increasing temperature and applied current density (Figure 1A). At 25°C, the CD_max_ was 50 mA/cm^2^ with deposit containing approximately 14.5 at% Sm (*i.e.,* 30.2 wt%). Increased solution temperature (60°C) extended the CD_max_ to 500 mA/cm^2^, resulting in the deposit Sm content of approximately 30 at% (*i.e.,* 55 wt%), sufficient for a series of stoichiometric SmCo intermetallic compounds (after appropriate annealing): Sm_2_Co_17_, SmCo_5_, SmCo_7_ and SmCo_3_. The current efficiencies (CEs) initially decreased sharply, leveling with CD exceeding 50 mA/cm^2^ (Figure 1B).

**FIGURE 2:**
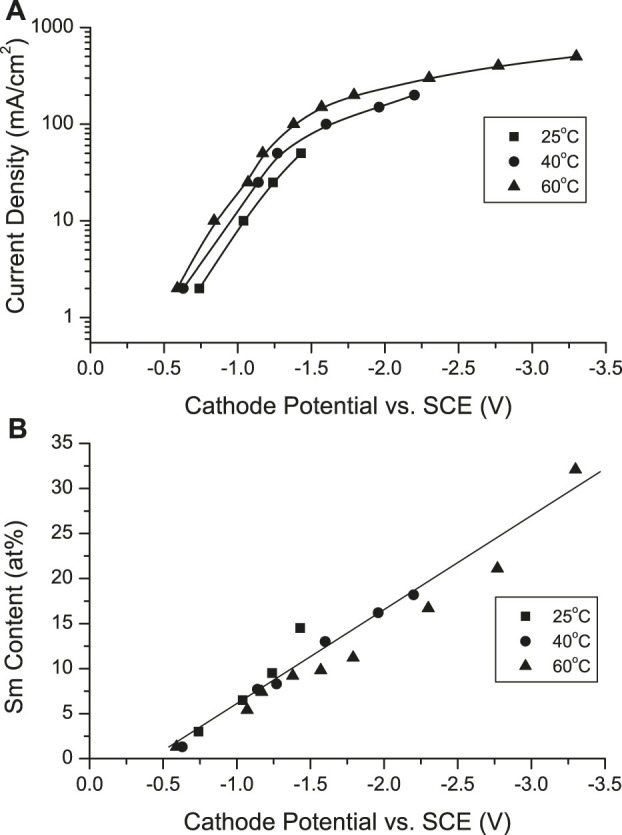
**(A)** Cathodic polarization curves in the electrodeposition of Sm-Co alloys at various current densities and solution temperature and **(B)** dependence of Sm content on cathodic potential.

While the cathode potential became more negative with increased CD, it was less negative with increased solution temperature (Figure 2A). The deposit Sm content increased linearly with more negative potentials, apparently independently of solution temperature (Figure 2B). However, co-deposition of SmCo initiating a potential less negative than the equilibrium potential of Sm (E^o^_Sm/Sm_^3+^ = −2.65 vs SCE) indicated a deposition mechanism involving a potential resulting from complexation rather than direct electrodeposition from aqueous ions.

**FIGURE 3:**
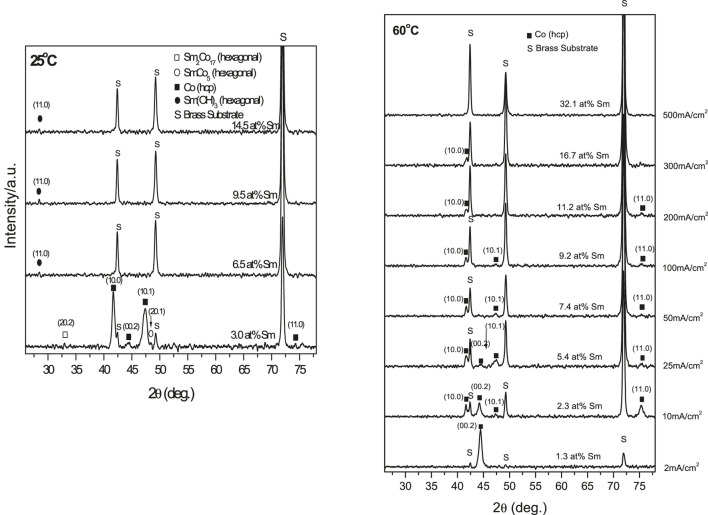
XRD patterns of electrodeposits obtained at different temperature (i.e., 25 and 60°C) and various CDs.

X-ray diffraction spectra (XRD) of Figure 1 deposits indicated structures changing from crystalline to noncrystalline (amorphous) with increasing Sm content (Figure 3). The crystolites consisted of α-Co phases (hexagonal close packed (hcp)) or Sm (rhombohedral) phases were observed. Deposits formed at 25°C were essentially amorphous with low Sm(OH)_3_ content. Low CD (2 mA/cm^2^, 3 at% Sm) deposit showed strong 10.0 and 10.1 *α*-Co (hcp) peaks and weak (20.1), (20.2) SmCo_5_ and Sm_2_Co_17_ (hcp) peaks, respectively (Figure 3).

**FIGURE 4:**
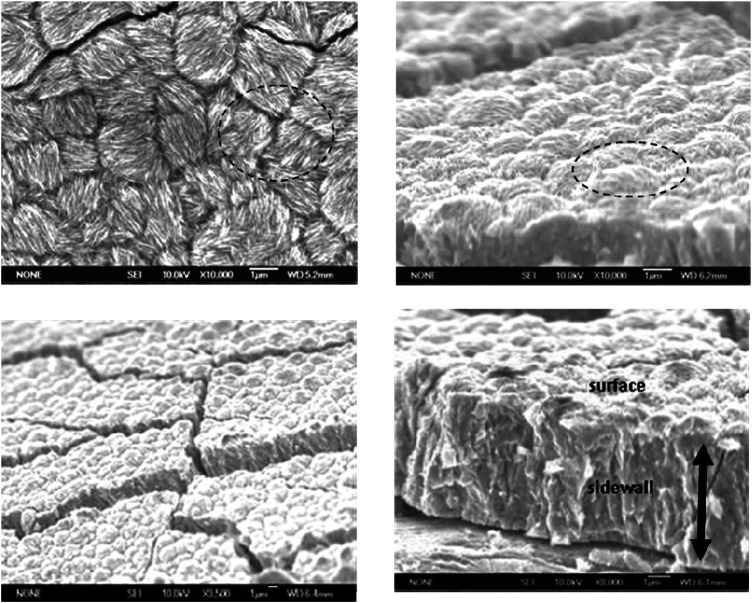
Top and cross-sectional scanning electron microscopic images of Sm-Co electrdeposits at different magnification; 9.2 at% Sm, ∼ 5 μm, pH 5.7, 60°C, 100 mA/cm^2^.

XRD spectra of 60°C electrodeposits (not shown) indicated a slight shift in the Bragg angles (α-Co 0.002 and 10.0 peaks) with increasing Sm content. Differing atomic radii of Co (1.25 Å) and Sm (1.81 Å) suggested a misfit, (RSm-RCo) = 0.45], which could result in Co lattice distortion, which tends to elongate the Co lattice while compressing it along the basic plane and likely generate residual stresses in the SmCo deposit contributing to microcracks (Figure 4).

Figure 4 shows the SEM images of SmCo alloys electrodeposited at 60°C and 100 mA/cm^2^, which revealed a cracked nodular surface. At higher magnification, fibrous nanorods with varying random orientation emanating from individual nodules (Figures 4B,C) were observed, as with other electrodeposited cobalt and cobalt alloys (Cavallotti et al., 1983). The estimated nodule diameters ranged approximately from 1.5 to 2.5 μm, the crack widths from 0.12 to 0.15 μm with an estimated density of approximately 1,000 cracks/cm^2^. The texture of the mechanically fractured sidewall was indicative of the deposit’s brittleness (Figure 4D). It is suggested that the deposit nanocrystalline or amorphous structure and columnar growth may be the result of coalescing or bundling fibers (Figure 4C). Experience with electrodeposits of chromium (Cr) and electroless nickel (Ni) indicated that fine-grained nanocrystalline or amorphous deposit surfaces generally contain nodules (Ruan and Schuh, 2008).

**FIGURE 5:**
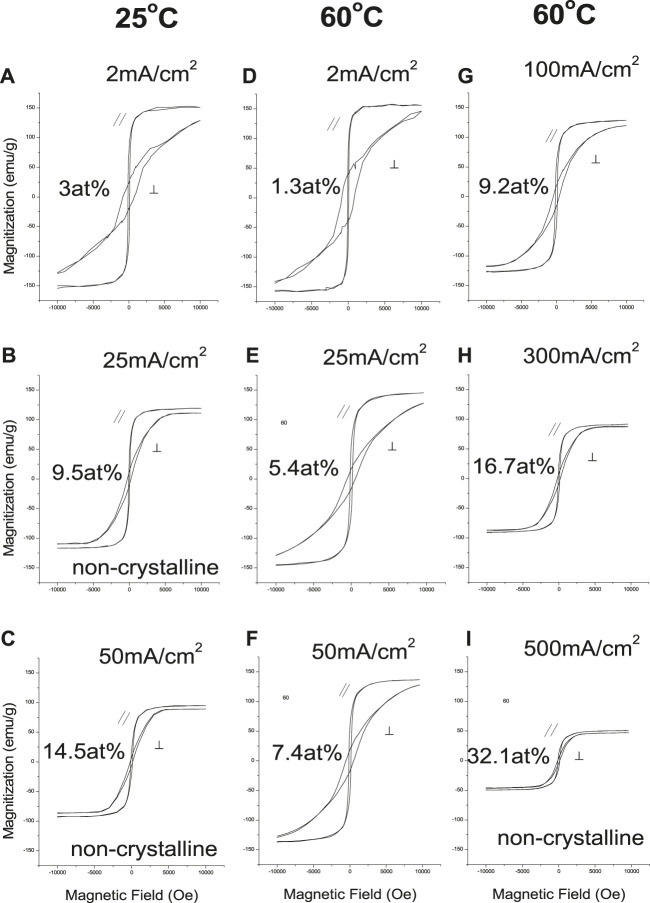
Magnetic hysteresis loops of Sm-Co deposits at 25 and 60°C, and various CDs.

**FIGURE 6:**
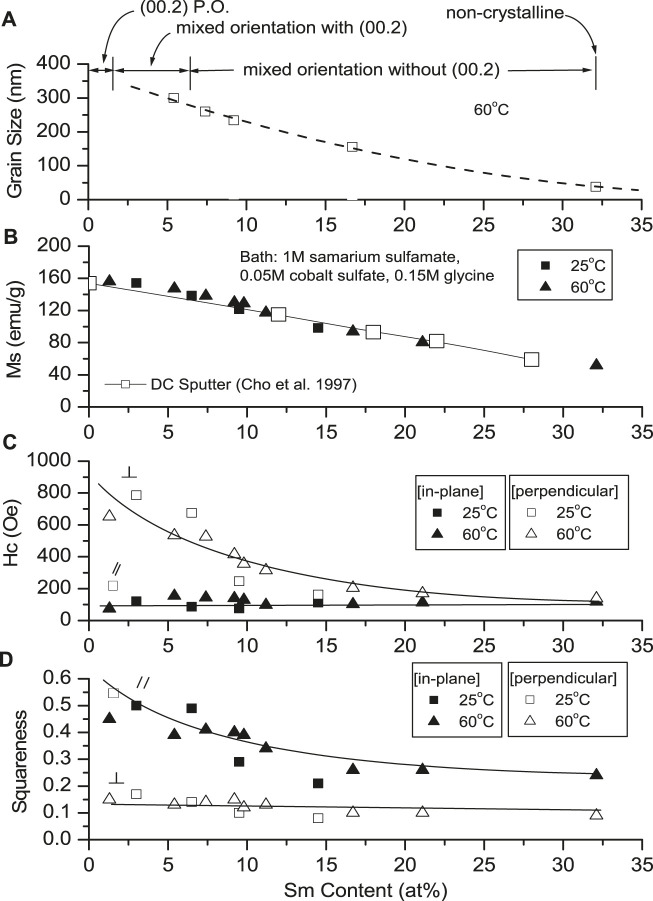
Effect of Sm deposit content and temperature on grain size and magnetic properties.

Figures 5, 6 show the effects of deposition variables on deposit composition and magnetic properties. Hysteresis loops indicated magnetic saturation (M_s_) was easier along the in-plane direction (easy axis) than the perpendicular direction (hard axis). The in-plane and perpendicular directions approached each other as deposit Sm content increased, while M_s_ and H_c_ decreased as CD increased. At constant CD and increased solution temperature (25°C–60°C), M_s_ and H_c_ increased, reflecting changing alloy compositions and structures (Figure 5)

Magnetization (M_s_) decreased linearly with increased deposit Sm content (Figure 6B), similar to sputtered deposits (Cho et al., 1997). Magnetic saturation of Co (M_s_ = 169 emu/g) (Bozorth, 1978) is higher than Sm (M_s_ = 0.3 emu/g) (Adachi et al., 1994), and the decreased M_s_ of the alloy was the result of decreased Co content. The deposit's structure changed from crystallinity to non-crystallinity with increased Sm content (Figure 6). Deposits with low Sm contents exhibited (002) plane orientation (*c*-axis), resulting in anisotropy.

As the deposit structure changes from crystallinity to non-crystallinity (increased Sm content), the deposits become more isotropic (Figure 5), and M_s_ and H_c||_ decreased. Deposits with low Sm contents exhibit 00.2 plane orientation (*c*-axis), resulting in anisotropy (H_c⊥_ >> H_c||_) (Figure 6C). Deposits with increased Sm have decreased hcp 00.2 peak intensity with decreased H_c⊥_. Deposits with high Sm content (32 at%) are non-crystalline and isotropic (H_c⊥_ ≈ H_c||_) as shown in Figure 6C with reduced M_s_ (Figure 6B).

Deposit coercivities in the in-plane direction (100 Oe) varied only slightly with deposit composition, but in the perpendicular direction, higher H_c_ (600–800 Oe) was obtained at low Sm content, decreasing sharply with increasing deposit Sm content. We note that as-deposited room temperature sputtered stoichiometric SmCo films also exhibited low coercivities (∼100 Oe), which did not increase substantially with subsequent annealing unless deposited on a Cr underlayer, promoting nanocrystalline c-axis texture in the SmCo deposit, increasing in-plane anisotropy (H_c_ > 40 KOe) (Prados and Hadjipanayis, 1998; Prados and Hadjipanayis, 1999).

In-plane coercivities remained constant regardless of the deposit Sm content; coercivity in the perpendicular direction, however, decrease with increased Sm content (Figure 6C). The squareness of the deposits appeared to be reversed (Figure 6D).

Deposit particle size decreased as Sm content increased, result of increased CD and/or decreased solution temperature (Figure 6A). Cavallotti *et al.* reported similar results for electrodeposited Co and Co alloys (Cavallotti et al., 1983).

**FIGURE 7:**
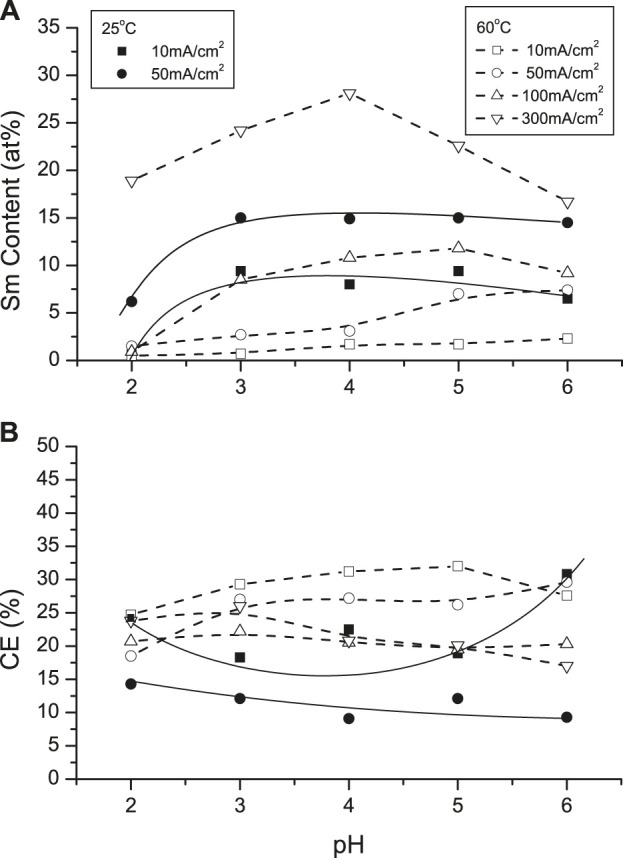
Effect of solution pH on **(A)** Sm deposit content and **(B)** current efficiency different temperature (25 and 60°C) and various CDs.

Figure 7A shows the dependence of Sm content on pH at various CDs (10 and 50 mA/cm^2^) at room temperature. Sm contents were higher than at 60°C but at the latter temperature maxima deposit Sm content were higher between pH 4 (12 at%, 100 mA/cm^2^) and pH 5 (28 at. %, 300 mA/cm^2^), respectively. Lower current densities and higher solution temperatures resulted in higher current efficiencies (CEs), but dependence on solution pH was not substantial (Figure 7B).

**FIGURE 8:**
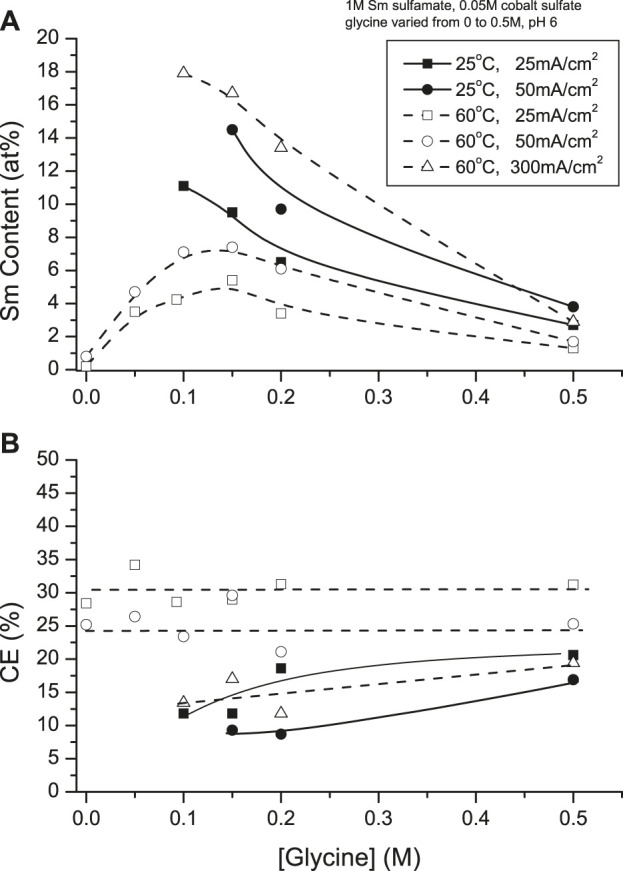
Effect of glycine concentration on **(A)** samarium deposit content and **(B)** current efficiency at 25 and 60°C.

Hull cell experiments (Wei et al., 2008) showed Co and Sm(OH)_3_ are electrodeposited from glycene-free solution, indicating a complex was essential for deposition of SmCo alloys. At 25°C, maximum deposit Sm contents were obtained at 25 mA/cm^2^ (∼11 at%) and 50 mA/cm^2^ (∼14 at%) with glycine concentration of 0.1 and 0.15 M , respectively; below 0.1 M, non-metallic deposits were formed. Increased solution temperature (60°C) and higher CDs resulted in increased Sm contents (*e.g.,* 300 mA/cm^2^, 0.1 M glycine to ∼18 at%) initially, decreasing with increased glycine concentration (>0.1 M). High deposit Sm contents were obtained with 2 to 3:1 glycine to Co ratios in the presence of excess Sm^3+^ (Figure 8A).

Current efficiencies (CEs) of 25°C deposits increased to 15% with increased CD and glycine concentrations. At 60°C, there appeared to be no significant CE dependence, although CE increased to 30% at higher CDs (Figure 8B).

XRD spectra (not shown) indicated that 0.15 M glycine containing solutions produced nanocrystalline or amorphous deposits with a weak 11.0 Sm(OH)_3_ peak at 25°C but none at 60°C. This confirmed observations of the HC studies (Wei et al., 2008), *i.e.,* glycine inhibited formation of hydroxides in agreement with Diven et al. (2003) that glycinato-Co complexes inhibit formation of Co(OH)_2_ in aqueous solutions. Interestingly, XRD spectra of deposits from solutions with 0.05 M or 0.5 M glycine concentrations show the presence of Sm(OH)_3_ and Co(OH)_2_ 11.0 peaks, which suggested an optimum concentration of complexant: M (metal ion) ratio, minimizing or inhibiting hydroxide/oxide inclusions. At 60°C, deposits also exhibited several Co (hcp) peaks which did not appear in 25°C deposit spectra.

**FIGURE 9:**
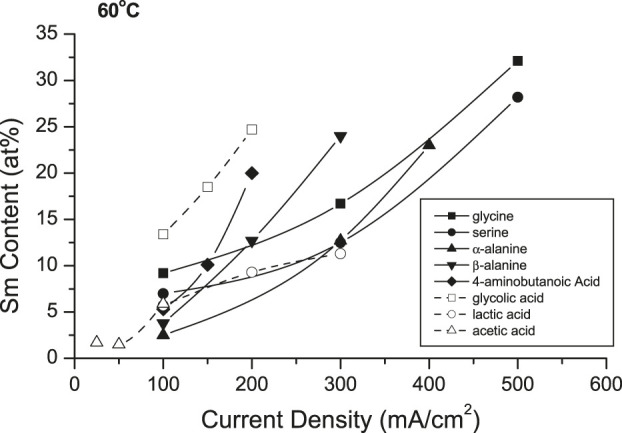
Effect of various complexes on samarium deposit content at 60°C (pH = 5.8).

Figure 9 shows the effects of selected complexants (0.15 M) on the deposit Sm contents. In 25°C solutions, only the amino acids appeared to be effective complexants (Table 1), while the other tested complexants resulted in burnt or powdery deposits containing hydroxides/oxides. Increasing solution temperature to 60°C resulted in extending the CD ranges and permitted co-deposition with other amino acids and hydroxycarboxylic acids, analogous to glycine and alanine (glycolic and lactic acids) with decreased CD_max_ (Table 1). Substitution of other complexants for glycine indicated glycine provided higher deposit Sm contents, but polycarboxylic acids (e.g., citric acid, EDTA) presumably resulted in stronger complexes which prevented deposition of SmCo alloys. These results suggested the bond strengths and/or structures of the various (aqueous) coordination compounds and their interdependence with the deposition variables are paramount in the co-deposition of the alloys, their compositions and magnetic properties.

**TABLE 1:** Summary of CD_max_ and max. Sm contents obtained from solutions containing different complexers.

	25^°^C		60^°^C	
Complexer	CD_max_ (mA/cm^2^)	Max. Sm content (at%)	CD_max_ (mA/cm^2^)	Max. Sm content (at%)
Acetic acid	nd	nd	100	6
Glycine	50	15	500	32
Serine	50	13	500	28
α-alanine	50	12	400	23
β-alanine	nd	nd	300	24
4-aminobutanoic acid	nd	nd	200	20
Glycolic acid	nd	nd	200	25
Lactic acid	nd	nd	300	11
Citric acid	nd	nd	nd	nd
EDTA	nd	nd	nd	nd

nd, non metallic deposit.

**FIGURE 10:**
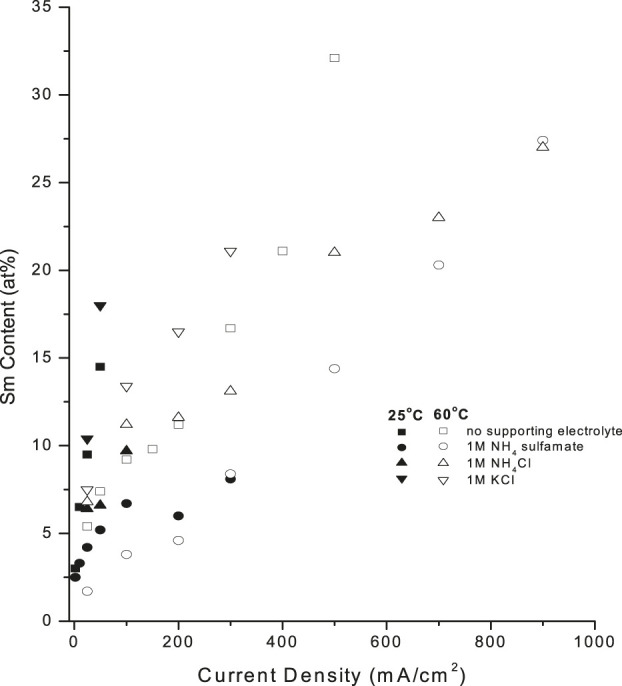
Effect of supporting electrolyte on samarium deposit content at **(A)** 25°C (closed pts) and **(B)** 60°C (open pts) [Note- draw continuous and dashed connecting line].

Supporting electrolytes (SE) are frequently added as solution components to increase solution conductivity, stabilize solution pH, and permit higher CDs, affecting deposit composition and properties. Figure 10 shows the effect of KCl, NH_4_Cl and NH_4_ sulfamate on deposit Sm content. At 25°C, KCl increased the deposit Sm content up to 18 at%, which was higher than that in the absence of supporting electrolyte (15–14 at%), while NH_4_Cl and NH_4_ sulfamate decreased the Sm content to 9.7 at% and 8.1 at%, respectively. CD increased substantially at 60°C in both the absence and presence of supporting electrolytes (Figure 10).

**FIGURE 11:**
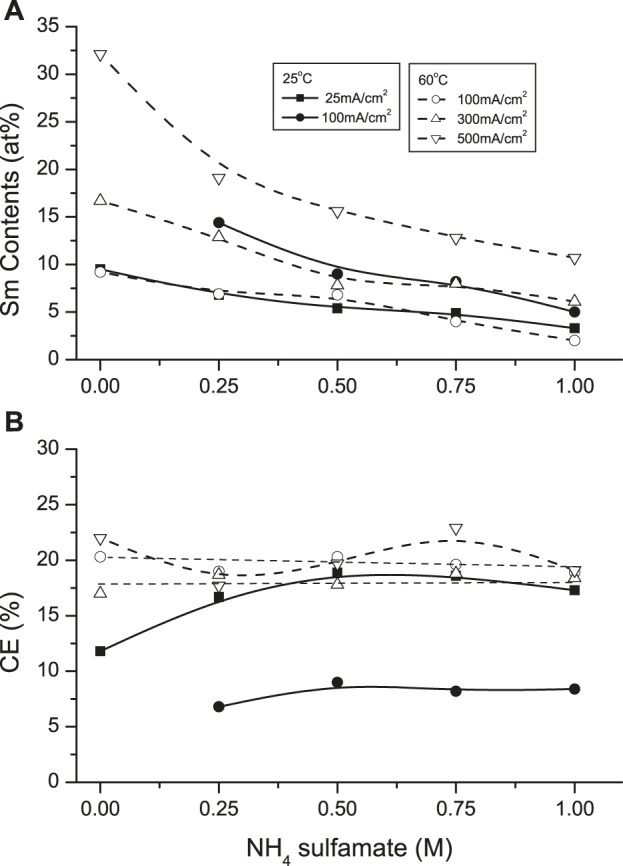
Effect of NH_4_ sulfamate concentration on **(A)** samarium deposit content and **(B)** current efficiency at 25 and 60°C.

Ammonium compounds are widely added to plating solutions as these SE may participate in complexation of the depositing ions. Ammonium sulfamate was investigated as SE in the SmCo solutions (Figure 11). Increasing additions of NH_4_^+^ (0–1 M) decreased the deposit Sm contents from solutions at both 25°C and 60°C. This effect was more pronounced at the higher temperature: Sm deposit contents decreased from ∼32 at. % Sm (no NH_4_^+^) to ∼11 at% Sm (1 M NH_4_^+^) at 500 mA/cm^2^, with similar decreases at lower CDs. This might be attributed to the deprotonation of ammonium (NH_4_^+^ → NH_3_ + H^+^), which could form other competing complexes such as cobalt hexammine ion (Co(NH_3_)_6_^2+^), favoring Co deposition. Furthermore, NH_3_ could modify the proposed heteronuclear-glycinato-complexes (Schwartz et al., 2004) with the inclusion of bridging NH_3_ ligands, which are not conductive to facilitate electron transfer in redox reactions (Taube and Gould, 1969).

### Proposed Deposition Mechanism

Coordination compounds, such as inorganic complexes (ligands)—cyanide, halide, hydroxide, and phosphate complexes—, have been employed in electroplating systems since the early 1800s and are increasingly involved in many commercial processes. Organic ligands including polycarboxylic, hydroxycarboxylic, aminocarboxylic and heterocyclic compounds are also well-known complexing agents for the electrodeposition of single metals and alloys from aqueous plating solutions. Tartrates, citrates, hydroxyacetates, hydroxypropionates and glycinates are extensively employed in electroless deposition and electrodeposition of alloys.

In addition to engineering, electronic, and magnetic applications of these electrodeposited alloys, there is increasing interest in applying compatible samarium (Torres et al., 2001; Torres et al., 2003; Kremer et al., 2005), vanadium (Tsaramyrsi et al., 2001; Kaliva et al., 2002a; Kaliva et al., 2002b), molybdenum and tungsten (Kiss et al., 1995; Zhou et al., 1999; Zhou et al., 2000; Zhang et al., 2003; Zhou et al., 2004; Kustin et al., 2007) coordination compounds in biological (physiological) systems. Citrate ions participate in essential physiological processes (e. g., Krebs cycle) and as natural chelator for various metal ions; compatible amino acid and peptide complexes may interact with bodily citrate fluids and independently have enhanced effects as active biological agents for metalloenzyme processes and oncological treatments.

Yukawa and coworkers stressed the relevance of the coordination chemistry of amino acids and peptides in understanding interaction of trace metals with enzyme and other biological systems in bioinorganic and medicinal chemistry (Komiyama et al., 2008).

Franklin considered possible effects of complexation on electrodeposition mechanisms and deposition rates including adsorption or inclusion of complexed ions or molecules, complexation resulting in catalyzing deposition rate through ion bridging or ion pairing (Franklin, 1987).

The following observations pertinent to the proposed deposition mechanism were considered: only metallic Co and Sm(OH)_3_ deposited from Sm-Co solutions. Complexation with glycine or other ligand is a required constituent for electrodeposition of Sm-Co alloys. The structure and geometrics of the complex, along with the deposition variables determined the deposition rates of both Co and Sm, the resultant alloy composition, grain size and other properties. The extensive industrial application of chromium plating was used for comparison (Hoare, 1989). Although the toxic Cr(VI) regularly electroplates to Cr, much less toxic Cr(III) cannot. Mandich reported that Cr(III) is a strongly hydrated ion, which precludes its electrodeposition to metallic Cr (Mandich, 1997). However, complexed with a suitable organic ligand, Cr(III) deposits to Cr (Danilov and Protsenko, 2001; Song and Chin, 2002).

**FIGURE 12:**
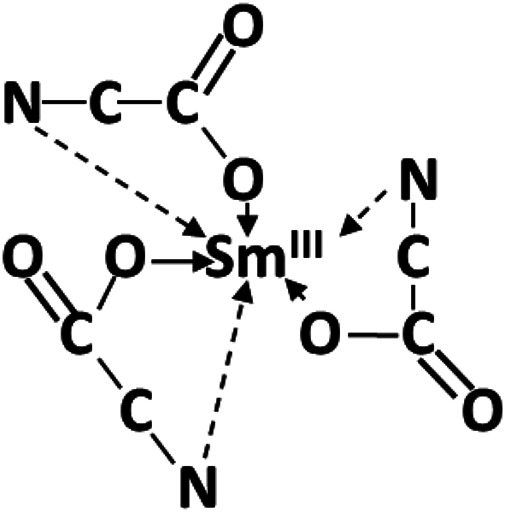
Sm glycine chelate complexes adapted from Prados and Hadjipanayis (1999).

Yukawa showed the versatility of glycine and other amino acids and peptides as complexing chelated molecules (Komiyama et al., 2008). The versatility of glycine is based, in part, on various protonation/deprotonation configurations. Sm glycino-complexes either as chelated monomeric Smgly_3_ (Figure 12) or dimeric coordinated compounds*,* i.e., complexes resulting in high stability constants, possibly preventing electrodeposition (Torres et al., 2001; Torres et al., 2003; Kremer et al., 2005).

**FIGURE 13:**
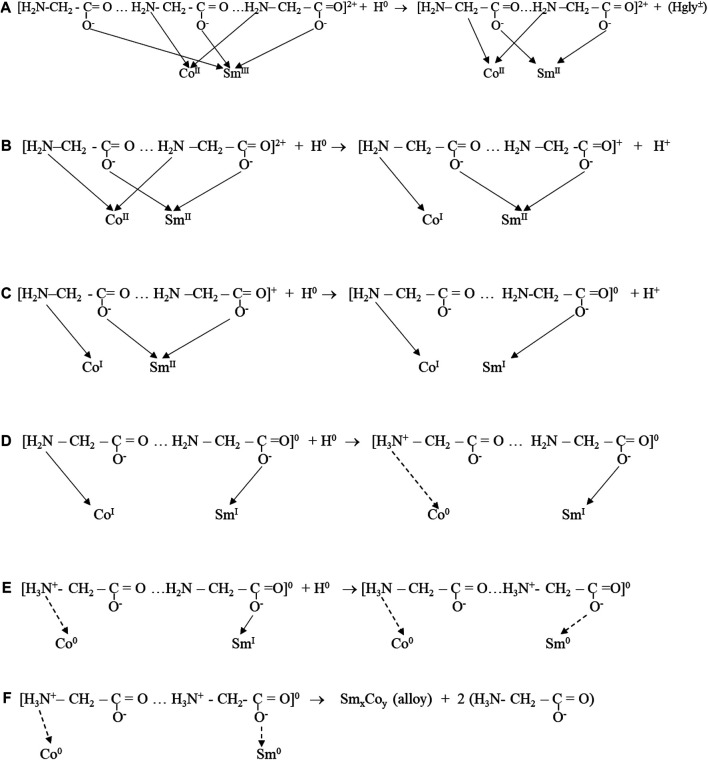
Incremental atomic H reduction of Sm-Co ions in glycine structure.

Zvaginsteva and Goncharov considered the polymerization of glycine as peptides (Zvaginstev and Goncharov, 1963). It is envisioned that quasi-peptides structures developed as a result of H-bonded bridges by O...H…N bonds. The presence of Co ions possibly inhibited Sm-glycine chelated complexes, resulting in catenated heteronuclear trisglycino- complexes coordinating cis oriented Co and Sm ions through the glycine carbonato- and amino- groups, respectively (Figure 13).

In gas phase catalysis, hydrogenation of organics usually proceeds by adsorbed hydrogen atoms on surfaces; this generally requires high temperatures and/or pressures as contrasted with aqueous phase hydrogenation (Ceyer, 2001). Further, in aqueous phase electrocatalysis, adsorbed hydrogen atoms readily reduce and hydrogenate organics and promote polymerization (Parravano, 1951; Park et al., 1985; Li et al., 2012).

**FIGURE 14:**
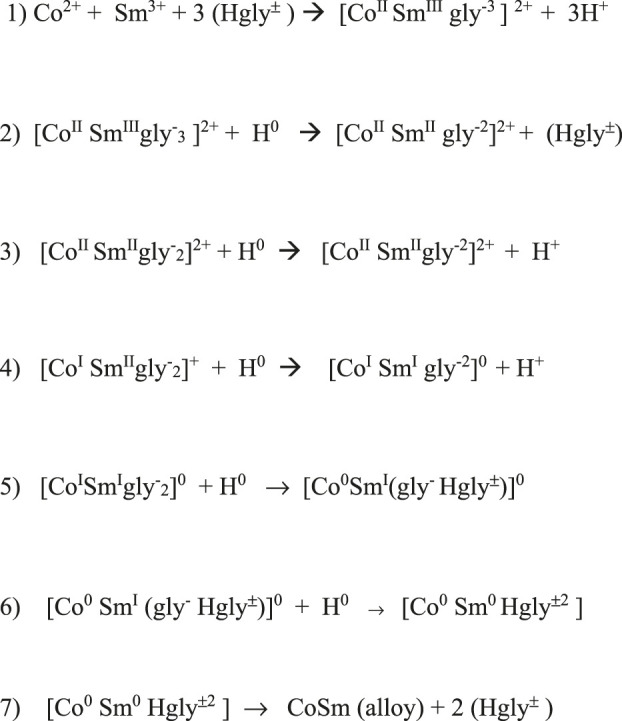
Proposed stepwise reduction of Sm-Co glycine complexes by H atoms.

H atoms generated and adsorbed at the cathode surface provided the electrons for the reduction and deposition of metal from the complex. The adsorbed hydrogen atoms reduced Sm^3+^ to Sm^2+^, modifying the complex. Continued stepwise reduction by hydrogen atoms resulted in zero-valent Co^0^ and Sm^0^ complex, which deposited on the electrode surface, resulting in an intimately mixed deposit constituting the equivalent of an alloy, Sm_x_Co_y_, with variable composition, depending on deposition conditions (Figures 13, 14).

Low CD deposition resulted in low Sm content and the presence of Co crystallites in the deposit. The reduction series of Co and Sm in the polymeric glycine- complexes and reaction flowchart (Figures 13, 14) show the suggested stepwise reduction process, culminating in the SmCo alloy.

## Summary

Samarium cobalt alloys were electrodeposited from aqueous solutions containing 1 M samarium sulfamate, 0.05 M cobalt sulfate, 0.15 M glycine, in presence and absence of supporting electrolytes. While they contribute to the solution stability, the supporting electrolytes used in this work decreased the Sm content in the deposit. Glycine or other coordination compounds were essential constituents in promoting co-deposition of Sm and Co; without complexing species, only Co metal and Sm hydroxide or oxide deposited. Glycine was a preferred ligand, resulting in higher deposit Sm contents while effectively inhibiting or minimizing occluded hydroxide/oxides in the deposit.

Polarization curves showed a linear dependence of deposit Sm content on cathodic potential with higher Sm content obtained at more negative potentials than the equilibrium potential of Sm, indicating complex species were involved in the co-deposition mechanism.

Increased solution temperature extended the CD_max_ from 50 mA/cm^2^ (25°C) to 500 mA/cm^2^ (60°C), resulting in high deposit Sm contents (32 at%), which satisfied the potential stoichiometric SmCo alloy compositions after annealing. The preferred solution pH range was between 2 and 6; pH > 6 resulted in nonmetallic deposits.

Magnetic saturation (M_s_) of deposits decreased with increased Sm content, becoming isotropic with deposits containing >30 at% Sm. Electrodeposited SmCo alloys and as-sputtered SmCo_5_ films exhibited low coercivities (i.e., H_c_ of ∼100 Oe as deposited).

Crystalline deposits became noncrystalline (amorphous) with increased deposit Sm content. Lower temperature and lower CD favor noncrystalline deposits with weak Sm(OH)_3_ peaks; no Sm(OH)_3_ peaks are observed in deposits from elevated temperatures.

A deposition mechanism involving the sequential stepwise reduction of the Sm and Co ions complexed with glycine (or other compatible ligand) by atomic hydrogen deposited at the cathode surface is proposed. Without complexation, only metallic Co and non-metallic Sm hydroxide/ oxide co-deposit.

## Data Availability

The original contributions presented in the study are included in the article/supplementary material, further inquiries can be directed to the corresponding author.

## Author's Note

This manuscript is dedicated to the pioneering research accomplishments of Dr. Ken Nobe and Mr. Morton Schwartz, who recently passed away. They were world-renowned electrochemical engineers and electroplaters known for their research on electrochemical processes including kinetics and mechanisms of electrodissolution, electrodeposition, corrosion, electrochemical energy systems, and bioelectrochemistry. This manuscript was the last manuscript they worked on together.
